# Endoplasmic reticulum and Golgi apparatus: old friends, novel intimate relationships

**DOI:** 10.1093/jxb/erx216

**Published:** 2017-08-21

**Authors:** Alessandro Vitale, Emanuela Pedrazzini

**Affiliations:** Istituto di Biologia e Biotecnologia Agraria, CNR, Milano, Italy, European Union

**Keywords:** Endoplasmic reticulum, Golgi apparatus, organelle laser trapping, protein traffic, secretory pathway

## Abstract

This article comments on:

Osterrieder A, Sparkes IA, Botchway SW, Ward A, Ketelaar T, de Ruijter N, Hawes C. 2017. Stacks off tracks: a role for the golgin AtCASP in plant endoplasmic reticulum–Golgi apparatus tethering. Journal of Experimental Botany 68, 3339–3350.

**The nature of the physical contacts between the endoplasmic reticulum and the Golgi apparatus has been debated for many years. Using advanced optical tweezer technology in living cells, Osterrieder *et al.* (2017) have now identified a Golgi membrane tethering protein that participates in anchoring Golgi stacks to the endoplasmic reticulum, opening the way to further functional studies of the role of these intimate relationships.**

The mid-nineteen century invention of subcellular fractionation and the application of electron microscopy to cell biology allowed us to discover the functional connections between the endoplasmic reticulum (ER) and Golgi apparatus in protein synthesis and secretion. This progress – which formed part of those steps forward resulting in the Nobel Prize for Physiology or Medicine in 1974 to Albert Claude, Christian de Duve and George Palade – opened the way to the discovery of intracellular membrane trafficking, the diverse compartments of the endomembrane system, and the secretory and endocytic pathways. The biosynthetic branch of the secretory pathway starts from the ER and leads to the Golgi apparatus as the first intermediate station. At the end of last century, the discovery of vesicle budding and fusion together with associated protein machinery, the continued refinement of electron microscopy, and the development of confocal microscopy and fluorescent protein tags – combining recombinant DNA and live imaging – have opened an intense and still-ongoing debate about the mechanistic aspects of the functional connections between compartments (Spang, 2013; Robinson *et al.*, 2015). This is particularly important at ER exit, where thousands of proteins destined for secretion or different endomembrane compartments start their life.

## Anchoring plant Golgi stacks

Golgins are protein tethers residing on the cytosolic side of Golgi membranes, and have emerged as key factors influencing membrane traffic to the Golgi apparatus (Gillingham and Munro, 2016). They capture vesicles, forcing them to collide on the target membrane. This is believed to favour membrane fusion at the compartment of destination, mediated by SNARE proteins, although golgin tethering is independent of SNARE activity (Wong and Munro, 2014). Golgins contain binding sites for Rab GTPases and coiled-coil motifs waving in the cytosol in search of contacts (Sinka *et al.*, 2008; Wong and Munro, 2014). It has therefore been clear that they could increase the efficiency of traffic at the ER–Golgi and endosome–Golgi interfaces, but whether they were directly responsible for the physical connection between the ER and Golgi apparatus had not been determined (Cheung and Pfeffer, 2016).

Building on the pioneering work describing the dynamic connections between the plant ER and Golgi apparatus (Boevink *et al.*, 1998), Osterrieder *et al*. (2017) have now determined for the first time that a golgin physically links these two compartments. The study takes advantage of optical tweezer technology and the specific features of the ER–Golgi system in plant cells. Optical forces coupled to microscopy can trap and move objects ranging in size from tens of nanometres to tens of micrometres (Grier, 2003). Optical tweezers have mainly been used *in vitro*, revealing mechanical and dynamic properties of cytoskeleton molecular motors or large biopolymers, while studies in living cells have been limited (Norregaard *et al.*, 2014). The Golgi apparatus comes in different forms, depending on the kingdom and even on species (Suda and Nakano, 2012; Brandizzi and Barklowe, 2013). The endocytic internalization of beads only a few micrometres across has been used to apply forces on mammalian Golgi membranes, but the entire mammalian Golgi apparatus – a single, perinuclear ribbon-like structure made of interconnected stacks – is too large to be trapped and moved (Guet *et al.*, 2014). Conversely, plant cells contain dozens/hundreds of individual stacks of Golgi cisternae, which constantly move along the ER tubules (Boevink *et al.*, 1998) and have an optimal size for optical trapping and displacement (Sparkes *et al.*, 2009). When the Golgi stacks of leaf epidermal cells are laterally displaced by optical tweezers, they drag ER tubules and can establish new contact sites at other ER locations (Sparkes *et al.*, 2009). Thus, the ER geometry can be manipulated by moving Golgi stacks. This can be performed after depolymerization of actin, indicating that the physical connection between Golgi and ER is not mediated by the cytoskeleton and is independent of Golgi movement (Sparkes *et al.*, 2009).

AtCASP (At3g18480) and two golgin-84 proteins (At2g19950 and At1g8190) are emerging as key golgins in Arabidopsis membrane traffic at ER exit sites (Renna *et al.*, 2005; Latijnhouwers *et al*., 2007). In the study by Osterrieder *et al.* (2017), overexpression of mutated AtCASP in which the cytosolic coiled-coil domains had been deleted (AtCASP-ΔCC) reduced the ER-dragging properties of Golgi stacks, thus implicating for the first time a specific protein in the *in vivo* physical connections between the two compartments. *In vivo* optical trapping of fluorescent protein-labelled compartments showed that AtCASP-ΔCC has dominant negative activity on the ability of the Golgi apparatus to drag ER tubules. This inhibition is not complete, strongly suggesting that other factors contribute to these Golgi–ER interactions. The authors also cautiously state that the demonstration of protein-mediated connections does not exclude the existence of possible membrane continuity between the ER and Golgi, which could participate in organizing the ER–Golgi interface, but the experiments clearly show that AtCASP physically links the two compartments.

## Role in trafficking

A question arising from the present study is whether functional traffic phenotypes originate from the weakening of Golgi–ER tethering caused by the overexpression of AtCASP-ΔCC. As already noted, thousands of newly synthesized proteins of the endomembrane system move from the ER to the Golgi apparatus, and from there they can then reach the distal compartments of the endomembrane system, be secreted, or be retrieved into the ER if they are part of the ER machinery. The fact that transgenic Arabidopsis expressing AtCASP-ΔCC is viable indicates that the secretory pathway is not severely compromised, which is consistent with the observation that the plant tethering system is redundant and the finding that the knock-out of the *Saccharomyces cerevisiae* CASP homologue COY1 does not impair yeast viability (Gillingham *et al.*, 2002). However, if tethering is important for traffic (Box 1), the partial effect of AtCASP-ΔCC on tethering could result in partial traffic defects. In their study, Osterrieder *et al*. (2017) have used GFP tagged with the C-terminal tetrapeptide HDEL (GFP-HDEL) to visualize the ER, and GFP or RFP tagged with the transmembrane domain of a Golgi resident to visualize Golgi stacks. The HDEL tetrapeptide is present in several soluble ER residents and actually functions as a retrieval system from the Golgi apparatus. Proteins with a C-terminal HDEL sequence (or its variant KDEL) do traffic to the Golgi apparatus, but are efficiently retrieved back into the ER by the KDEL/HDEL receptor, which is located mainly in the *cis*-Golgi cisternae and moves back into the ER upon ligand binding (Gomez-Navarro and Miller, 2016). Neither the localization of GFP-HDEL nor that of the Golgi markers changes in the plants that express AtCASP-ΔCC, indicating that the dominant negative golgin does not have easily detectable effects on forward or retrieval ER–Golgi traffic. Full inhibition of protein traffic from the ER would be incompatible with eukaryotic life, but measurements on changes in traffic kinetics can be performed to detect quantitative effects, either using fast recovery after photobleaching or pulse-chase labelling and immunoprecipitation (see, for example, Tolley *et al.*, 2008). Besides those used by Osterrieder *et al.*, several other marker proteins that reach vacuoles or the plasma membrane, or are secreted, could be tested in searching for specific or subtle defects in traffic.

**Box 1. Two possible models for putative AtCASP function in protein trafficking**

ER–Golgi traffic could occur via COPII-coated tubules or vesicles (left or right part of each cartoon, respectively). In model (A), all secretory proteins benefit from golgin AtCASP tethering in exiting from the ER. The stabilization of the ER–Golgi membrane connections by AtCASP is not strictly necessary, but could increase the efficiency or kinetics of traffic. Tethering of the ER membrane is mediated by the AtCASP cytosolic, N-terminal coiled-coil domain. The binding partner at the ER exit sites could be a Rab protein. In model (B), AtCASP tethers and stabilizes the connection between ER and Golgi membranes to facilitate trafficking only of specific proteins. Trafficking of other cargoes does not depend on AtCASP function.

**Figure:**
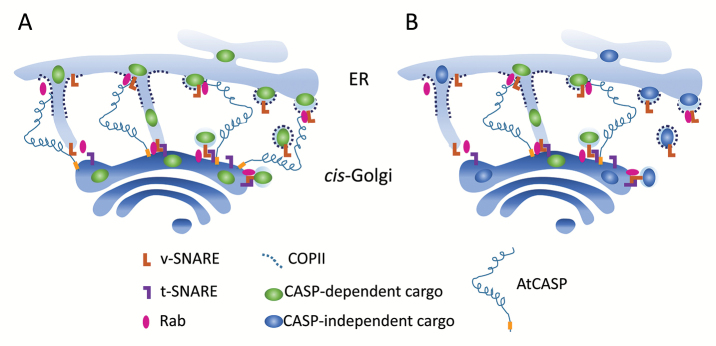

It should also be considered that most golgins are peripherally anchored to membranes, but CASP and golgin-84 are anchored to the *cis*-Golgi membrane via a C-terminal transmembrane domain and are thus tail-anchored (TA) integral membrane proteins (Gillingham and Munro, 2016). AtCASP contains predicted putative nuclear and chloroplast targeting signals which caused its exclusion from published TA-catalogues, whereas golgin-84 was correctly predicted as a TA protein (Kriechbaumer *et al*., 2009; Pedrazzini, 2009). TA proteins resident on the endomembrane system are not directly targeted to the membrane of destination: they are first inserted into the ER membrane and from there they traffic to reach the Golgi. Therefore, TA-golgins are at the same time both tethers and cargoes. Their TA transmembrane domain could be an intra-bilayer sensor of the ER–Golgi traffic interface and could be involved in the selection of unknown specific cargo. In this respect, it has been observed that point mutagenesis of a conserved histidine in the Coy1p transmembrane domain affects activity of this golgin without any apparent effect on its localization (Gillingham *et al.*, 2002). As the authors underlined, this suggests that the Coy1p transmembrane domain, and possibly that of its mammalian and plant homologues, has additional, as yet unknown, function(s) besides attaching the protein to the membrane. Whatever the actual role played by AtCASP in secretory traffic, the identification of an ER–Golgi connecting protein represents a milestone in our knowledge of the architecture and dynamics of the endomembrane system, and opens the way to a full dissection of its tethering mechanisms.
